# The Opium Treatment of Peritonitis1Read at the fortieth annual meeting of the Medical Association of Central New York, held at Rochester, October 15, 1907.

**Published:** 1908-02

**Authors:** Charles G. Stockton

**Affiliations:** Buffalo, N. Y.


### BUFFALO MEDICAL JOURNAL.

VOL. LXIII. FEBRUARY, 1908. No. 7

INDEX THROUGH

### ORIGINAL COMMUNICATIONS.

### The Opium Treatment of Peritonitis[1]

BY CHARLES G. STOCKTON, M. D., Buffalo, N. Y.

I have been requested to state my views concerning the treatment of general septic peritonitis. This is probably owing to the fact that it has been my habit for a number of years to teach my pupils that I was convinced that the use of opium in the treatment of general peritonitis, as formerly originated and advocated by Alonzo Clark, had fallen too much into disuse. While I am prepared to defend this position, I wish in the beginning to be distinctly understood not as advocating the opium treatment to the displacement of surgery in general septic peritonitis. There can be no reasonable doubt but that when the abdominal cavity practically takes on the character of an abscess it should be treated as an abscess along surgical lines, with evacuation, drainage and cleanliness.

I have never taught that the opium treatment is especially suited to septic peritonitis as compared with other types of peritoneal inflammation. On the contrary, I believe that the greatest usefulness of the opium treatment belongs to those milder cases before the inflammation has become general in the abdominal cavity. The grounds on which these views are based are, first, those of clinical observation; and, second, on the results of physiological experiments.

*Clinical Observation.*—There are yet men living who had the opportunity of observing acute general peritonitis as it progressed under the treatment originally advocated by Alonzo Clark. The majority of the profession have either forgotten this treatment, or else have misinterpreted its nature. The most available source of learning the precise views of Alonzo Clark may be found in his classic article appearing in "A System of Practical Medicine by American Authors," generally known as "Pepper's System,"

1. Read at the fortieth annual meeting of the Medical Association of Central New York, held at Rochester, October 15, 1907.

published in 1885. Previous to the appearance of this article Clark's treatment was falling into disuse, and it has appeared to me difficult to understand the technic of his procedures without learning the method under the eyes of those who have seen it properly carried out. As a student and young practitioner I had the opportunity of learning the treatment from Dr. Thomas D. Strong of Chautauqua County, and from Dr. Thomas F. Rochester, my honored predecessor in the University of Buffalo.

Clark taught that as soon as the diagnosis was made the largest safe dose of opium should be administered. He aimed to use very large doses with long intervals between, yet never allowing the patient to emerge from the complete sedative effects of the drug. In general peritonitis the amount of opium and morphine that can be taken safely is remarkable. Two or three grains hypodermatically are required during the height of the disease, and I have occasionally injected five grains at intervals of from four to six hours, for several days in succession. The aim of the treatment is to keep the patient as quiet as possible, and yet within the borderland of safety. To one experienced with the drug in this disease, there is little danger of over use. As the inflammation subsides the tolerance for opium decreases and the dosage is lessened, and very soon it is unnecessary to administer any anodyne whatever.

Under its effects the respirations fall to eight or ten a minute, the pupils contract, the skin becomes warm and moist, the patient scratches his nose on awakening, lies with legs extended, the abdomen is soft, but the functions of the body are wonderfully little disturbed. The evacuations of the bladder and bowels are more likely to occur spontaneously with the opium treatment than without it. The failure of the treatment, in my judgment, usually results from timidity in dosage resulting, first, in increased tension of the pulse, then the reappearance of pain and the rigidity of the abdomen, all of which necessitate larger and more frequent doses than should have been necessary in order to relieve the symptoms. Each time the patient comes out from the sedative influence of the drug, the disease seems to make appreciable headway; whereas, with the steady sedative effects carefully maintained, the disease does not proceed but, as a rule, is controlled and recovery takes place.

I have seen too many serious cases of general peritonitis go on to recovery under the opium treatment not to feel confident in the truthfulness of the claims put forward by Alonzo Clark, and by Sands, Smith and others of his students. It never could succeed in inexperienced or timid hands. The moderate use of opium only results in covering up symptoms, never in curing patients. The fatal blow to the popularity of this treatment was given by the teachings of Lawson Tait. There can be no doubt of his wonderful results, and he insisted that not a particle of opium should be given.

*Experimental Studies.*—Now, as to the reason for the success of the opium treatment in peritonitis. In the first place, to a large extent, it controls abdominal shock. The experiments of Crile have demonstrated that opium is an important agent in preventing and overcoming shock. Under its effect the blood pressure rises to a safer point. I believe that herein may be found one of the reasons for the usefulness of opium in peritonitis. Again, the intra-abdominal circulation is favored by opium which overcomes the spasm of the abdominal parietes and the spastic state of the vessels. Further, it is shown that marked "stimulation of the splanchnic nerves causes complete relaxation of both coats of the small gut." . . . . "It seems that the splanchnics normally exercise a tonic, inhibitory influence on the intestinal movements." . . . . . "On this account it is often impossible to obtain any movements in the exposed intestine (in experiments) so long as this remains in connection with the central nervous system by means of the splanchnic nerves." . . . . . "The relaxed condition of the gut which obtains in many abdominal affections is probably also reflex in origin, and is but a reflex inhibition through the splanchnic nerves." These sentences are quoted from Starling's recent work "The Physiology of Digestion." 1906, p. 143.

Thus, modern physiology explains how tympanitis occurs in peritonitis, and it shows not only how this symptom may be overcome by the action of opium, but also why it is that shock is reduced through the sedative influence of opium. Undoubtedly it would be better to remove the cause of irritation of the splanchnics, and hence I have no argument against surgical treatment of peritonitis, so long as it is employed at the right time and by the right man. On the other hand, I fully believe that there is an important and neglected field for the employment of opium, and I conceive that lives are lost that might be saved by the use of opium. The surgeons insist that opium complicates the situation and obscures the field. This cannot be absolutely denied. We cannot turn away from the reasonableness of, nor from the necessity for, surgical treatment. I only plead the importance of the understanding of the nature of the opium treatment and for its application in a proportion of cases, a few of which are unsuited to surgery, and others in which it might prove an important accessory to surgery.

Opium is employed by a proportion of surgeons for the relief of pain in as small doses as possible. Some years ago a method for the use of the drug was advocated by Ocshner, and his plan met with adherents, but the manner in which the drug was used is unlike that known as the Clark treatment. Recently Professor Pel of Amsterdam (*Berl. Klin. Woch.*, August 12, 1907), advocated the moderate use of opium for the alleviation of pain and for the lessening of other symptoms. His article is interesting and he points out that in peritonitis opium does not constipate nor lessen elimination. The chief advantages which he attributes to the drug is in facilitating rest, localising morbid processes by suppressing the peristaltic movements, thus preventing the breaking of adhesions. It will be observed that this method of employing the drug is quite unlike that advocated by Alonzo Clark. It would seem to possess most of the objections which might be urged against the Clark treatment, and yet it lacks the cardinal advantage of inducing a complete sedative effect and continuing this until the inflammation has subsided and the pus walled off by adhesions.

Undoubtedly in most cases of septic peritonitis, the drainage of one or more abscess cavities would ultimately become necessary; but this means a comparatively small incision made at a time when an operation is practically free from danger. In cases of septic peritonitis early recognised, such, for instance, as that so frequently observed in acute appendicitis, an immediate operation I believe to be the only proper procedure; but in cases that are neglected and where manipulation of the abdominal viscera is likely to extend the peritonitis even though the appendix is successfully removed, it appears to me safer to resort to the Clark treatment, waiting for a more favorable opportunity to operate.

Owing to the acute activity of my surgical colleagues and to the diagnostic acumen of the general practitioners, I now rarely have the opportunity of recommending a course of management with which I was at one time very familiar. I am not prepared to admit that the results are better now than they were then, provided that an operation for drainage was made at the right time, thus obviating the dangers and embarrassments that are likely to follow upon prolonged suppuration.

The last case that I recall was in my ward at the Buffalo General Hospital. It was that of a little girl about eleven years old, who entered after several days illness of acute peritonitis depending upon neglected appendicitis. She was in excruciating pain and manifested the usual evidences of abdominal shock. The treatment consisted in morphine administered hypodermatically. The first dose given was one-third of a grain, two hours later one-half grain was administered and repeated, and the next day one grain was injected about once in four hours. Ultimately she had doses of two to three grains at intervals of from four to six hours. From the first she was kept in a state of perfect calm, free from pain, sleeping quietly, and at the expiration of a week it was necessary to reduce the morphine to minute doses; at the expiration of a fortnight an abscess in the right iliac region was drained, and the girl made a prompt and complete recovery. I am convinced that the child would have succumbed if a laparatomy had been performed at the time of her admission to the hospital.

There is nothing extraordinary about this case, and yet some of the onlookers regarded the amount of morphine injected as dangerous and uncalled for. I think any smaller amount would have been dangerous, and that the use of the drug simply to moderate pain and obscure symptoms without producing a complete sedative effect, is almost to be regretted.

If I have made myself understood, you will perceive that I believe that the proper treatment of oncoming septic peritonitis is immediate operation. but that when the operation is delayed and the inflammation is becoming general, it is safer to use the full opium treatment until such time as the suppuration is localised and drainage effected without molesting the greater part of the abdominal cavity.

436 FRANKLIN STREET.

